# Factors associated with HIV testing and intention to test for HIV among the general population of Nonthaburi Province, Thailand

**DOI:** 10.1371/journal.pone.0237393

**Published:** 2020-08-14

**Authors:** Patou Masika Musumari, Teeranee Techasrivichien, Kriengkrai Srithanaviboonchai, Arunrat Tangmunkongvorakul, Masako Ono-Kihara, Masahiro Kihara

**Affiliations:** 1 Global Health Interdisciplinary Unit, Center for the Promotion of Interdisciplinary Education and Research, Kyoto University, Yoshida-honmachi, Sakyo-ku, Kyoto, Japan; 2 Research Institute for Health Sciences, Chiang Mai University, Sriphum, Muang Chiang Mai, Thailand; 3 Department of Health Informatics, Kyoto University School of Public Health, Yoshida Konoe-Cho, Sakyo-ku, Kyoto, Japan; 4 Department of Community Medicine, Faculty of Medicine, Chiang Mai University, Chiang Mai, Thailand; National University of Singapore, SINGAPORE

## Abstract

**Background:**

Research on HIV testing in Thailand has largely focused on at-risk population groups, with limited information about the prevalence of correlates of HIV testing among the Thai general population. This study addresses this gap in research by using a population-based probability sample to examine correlates of HIV testing experience and intention to test.

**Methods:**

We conducted a cross-sectional survey in Nonthaburi, Thailand during October-December 2012 using tablet computers to collect self-administered questionnaires from 2138 men and women (aged 15–59 years) identified through three-stage stratified cluster sampling.

**Findings:**

Almost half of the respondents, 962 (45%), reported having been tested for HIV while an almost equal proportion, 1032 (48.3%), indicated their intention to test for HIV. Being sexually experienced, having a history of sexually transmitted infection, personally knowing someone infected with HIV, and youth were associated with both history of HIV testing and intention to test for HIV. High perceived risk of HIV, knowledge of an HIV testing location, and having been married were associated with having been tested for HIV. Having been tested for HIV and HIV/AIDS education were associated with intention to test for HIV. The most common reasons for testing were routine medical checkup and antenatal care. The major reasons for not testing were perception of having no or low risk.

**Conclusion:**

A substantially low proportion of the respondents reported a history of HIV testing and intention to test for HIV. Culturally appropriate programs that address HIV risk perception and provide accurate information related to HIV infection and HIV testing may be beneficial in increasing uptake of HIV testing among the general population in Thailand.

## Introduction

HIV infection remains a leading global health priority, despite significant breakthroughs and progress in prevention and treatment over the past three decades [1]. The HIV epidemic in Thailand, as in many Southeast Asian countries, largely remains concentrated in higher risk subgroups, including men who have sex with men (MSM), sex workers, people who inject drugs (PWID), and transgender people [2]. Increased sexual risk in the general population represents a cultural shift among Thailand’s youth, increasing the possibility that the epidemic may spill over into the general population in the near future if unchecked [3–5]. Changes in sexual norms among Thailand’s youth include earlier sexual debut for both males and females, more lifetime sexual partners, and greater acceptance of adolescent premarital sex [3–5]. Correspondingly, the rates for unintended pregnancies and sexually transmitted infections (STIs) among Thai adolescents over the past 15 years [6, 7] have similarly increased. It is therefore critical to monitor the HIV epidemic in the general Thai population through effective and widespread HIV testing programs.

HIV testing is an essential gateway to both HIV prevention and treatment. There is evidence that awareness of one’s HIV status is correlated with a substantial decrease in high-risk behaviors [8, 9]. HIV testing promotes linkage to care and treatment, allowing individuals and the larger population to benefit from the preventive and health benefits of antiretroviral treatment (ART) [10, 11]. HIV testing is free and anonymous in Thailand [12, 13], and since 2012 adolescents younger than 18 no longer need parental consent to test for HIV [14]. Thailand’s National Operational Plan for Ending AIDS 2015–2019 utilizes the “Reach-Recruit-Test-Treat-Retain” framework as a means for advancing the country’s efforts to end the HIV epidemic [15]. With respect to the 90-90-90 target set by the Joint United Nations Programme on HIV/AIDS (UNAIDS), Thailand has achieved the first “90” with 94% of people living with HIV in 2018 aware of their status [16]. This highlights successful efforts to improve HIV testing and counseling in the country.

Research on HIV testing in Thailand has mostly focused on at-risk population groups, reflecting the concentrated nature of the epidemic in the country [17–19]. National population surveys, such as the 2006 National Sexual Behavior Survey and the 2009 Fertility Survey [5, 20], as well as localized research [21–23], have found HIV testing rates ranging from 18% to 48%. Self-efficacy, perception that HIV testing locations were easy to find, having two or more lifetime sexual partners, and history of pregnancy were associated with previous HIV testing among young Thais receiving non-formal education in Chiang Mai City [22]. Population-based studies in other countries have identified a range of factors independently associated with HIV testing, including higher level of education, higher wealth index, history of marriage, being older, HIV-related stigma, number of lifetime partners, and perceived quality of health services [24–26].

Population-level data has not made clear what factors influence HIV testing in Thailand. To address this gap, we use data from a population-based probability sample of an urbanizing province in Thailand. This study specifically aims to: 1) document the prevalence and correlates of HIV testing rates and 2) examine the factors that influence intentions to test for HIV among the general population in Thailand.

## Methods

### Study design, participants, & setting

**Fig 1:**
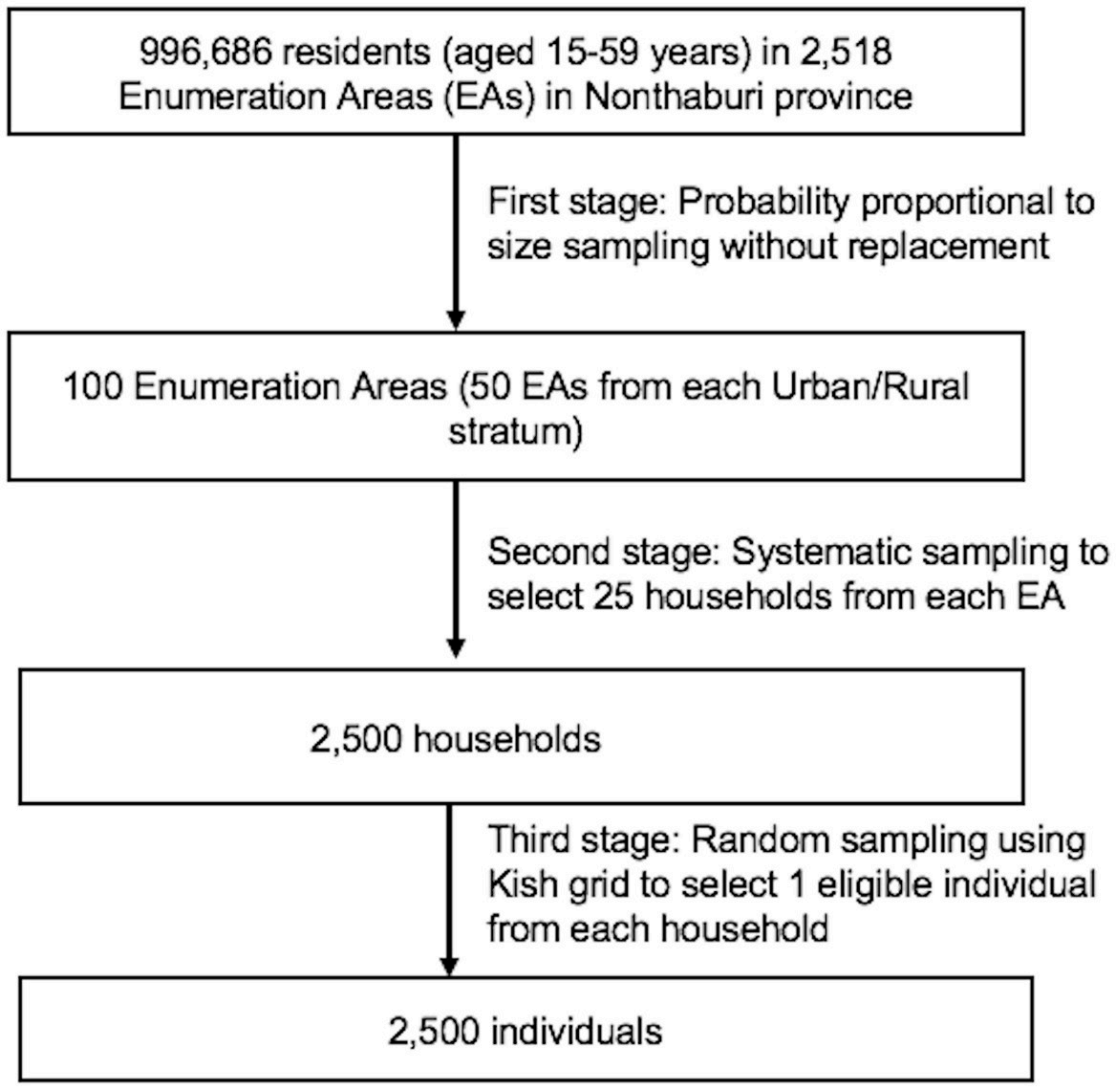
Sampling procedures.

This cross-sectional survey was conducted from October-December 2012 in Nonthaburi Province, in central Thailand. A detailed description of the sampling method is described elsewhere [3] and presented schematically here in Fig 1. In brief, we employed three-stage, stratified, probability proportional to size (PPS), clustered sampling to recruit 2138 men and women aged 15 to 59 years. In the first stage, 100 enumeration areas (EAs) (50 from each urban and rural stratum) were systematically selected by PPS sampling without replacement based on the 2010 National Population and Housing Census for Nonthaburi province. In the second stage, 25 households were then selected by systematic sampling from each EA from the list of all eligible households. The final stage consisted of the selecting participants from each of the sampled households using a Kish grid which gives nearly equal probability of selection to each household member [27]. This process was facilitated by a list of all eligible members within each selected household created by the field staff during household visits. [3].

### Ethical consideration

The research protocol was approved by the Committee for Research on Human Subjects of Kyoto University, Japan (E1320), and The Committee for Research Ethics (Social Sciences) of Mahidol University, Thailand (2012/072.0103 [B2]). All participants provided verbal informed consent prior to answering the questionnaire. Separate parental consent was obtained for respondents under 18. Following the questionnaire, respondents received HIV/AIDS-related educational pamphlets in appreciation for their participation.

### Data collection

Data were collected using a self-administered structured questionnaire based on a review of the Thai and international literature. A focus group discussion involving 20 local participants provided feedback to improve the initial draft of the questionnaire. The questionnaire was delivered through an internet-enabled tablet computer, designed to be user-friendly. The questionnaire was assessed for test–retest reliability over a 2-week interval in another set of 30 Nonthaburi residents. Kappa coefficients were calculated for dichotomous variables and intra-class correlation coefficients for non-dichotomous variables [28, 29]. All variables demonstrated good reliability ranging from 0.60–1.00. Lastly, we carried out the final pretest of the questionnaire among a separate set of 40 local residents to test for skip logic and final flow of the software. All individuals who participated in the instrument development phase were recruited from locations outside of our designated sampling areas and were not included in the main survey.

Fieldwork for the data collection was carried out by 14 field staff who had at least bachelor’s degree and prior field survey experience. All field staff attended a 1-week intensive training to learn about the study objectives and methods, research tools, how to approach potential participants, how to ensure confidentiality in participation, and informed consent. Data collection took place inside participant homes or somewhere nearby based on participant preference. All participants were allowed to complete the questionnaire in privacy, while field staff waited close by to assist if needed. The questionnaire software was programmed to automatically upload the results to the main server in real-time. Field staff had no access to the responses [3].

### Description of the variables

#### Dependent variable

The main outcomes of this study were: 1) prevalence of HIV testing, measured with the item “Have you ever been tested for HIV” and 2) intention to test for HIV, measured by “If there was an opportunity for HIV testing, would you like to be tested?”

#### Independent variables

Perceptions of risk were assessed at the country, interpersonal, and individual levels. Country level data were gathered using one multiple choice question, “Do you think HIV infection is decreasing, stable or increasing in Thailand?” from which participants chose decreasing, stable, increasing, or not sure/do not know. Interpersonal data were gathered through one question, “Do you personally know someone who has HIV/AIDS?” Individual level data included the question, “How likely are you to be infected with HIV?” with the following response options: unlikely, somewhat unlikely, likely, highly likely, and not sure/do not know.

Other covariates included socio-demographic information (age, gender, education, employment, marital status, and residential area), HIV risk status (sexual history, history of STIs), HIV-related knowledge and information (knowledge of where to get tested; awareness that HIV testing is free and anonymous for Thai nationals; exposure to HIV/AIDS-related information in the past 12 months; and knowledge of HIV transmission and treatment). Knowledge of HIV transmission, care and treatment was assessed with 12 items. Correct items for knowledge questions received a score of 1 while incorrect or unanswered questions received a score of 0. Scores ranged from 0 to 12. We used the median value of the score to categorize participants (< 9 and ≥9). A detailed description of the twelve knowledge items is provided in S1 Table.

We also explored reasons that participants opted to get, or not get, tested for HIV to get a contextual understanding for our analysis. Participants with a history of HIV testing, were asked, “What were your main reasons for being tested in the past?” Options included: personal or relationship reasons, part of a medical checkup or procedure, required for non-medical reasons, and other. Those who have never been tested were asked to choose among the following four options as reasons for not being tested: unlikely exposed, fear of HIV-related stigma, barriers to access, and other. We assessed facilitators of HIV testing with the question, “What factors would make it easier for you to get an HIV test?” Response categories included: easily accessible, free, anonymous, testing located far away where no one would recognize me, and other.

### Statistical analysis

Descriptive statistics were used to examine participant characteristics, reasons for getting tested for HIV, reasons for not getting tested, and facilitators of HIV testing. Chi-square tests and logistic regression models were performed to assess factors associated with previous HIV testing and intention to test for HIV. For each outcome, covariates associated with either previous HIV testing or intention to test for HIV as identify by a bivariate analysis with p-values <0.20 (to capture potential confounder) were included in the multivariable model. We included “Have you ever been tested for HIV” as a covariate in the HIV testing intention model as testing intention is largely recognized to be influenced by past HIV testing experience. All the analyses were performed using the Complex Sample module of SPSS for Windows version 17. Crude and adjusted odds ratios (OR) were reported along with their 95% confidence intervals (CIs).

## Results

### Sample characteristics

In total, 2,138 participants were recruited out of the 2500 people who were approached, yielding a response rate of 85.5%. The sample was composed of 1010 men (47.2%) and 1128 women (52.8%). The mean age of participants was 32.9 years (SD = 12.9) for men and 35.3 years (SD = 12.1) for women. Overall, 84% had at least a secondary school education, 81.1% were employed, 57.6% were married at some point, and 53.0% lived in a rural area. Close to half of participants either were tested or intended to get tested, 962 (45.0%) and 1,032 (48.2%), respectively. Just over half of the participants (53.4%) were not aware that Thai nationals were eligible for free and anonymous testing under the universal health care coverage scheme. (Table 1) Socio-demographic and behavioral characteristics of the participants are presented according to intention to test in S2 Table.

**Table 1:** Socio-demographic and behavioral characteristics of the participants by HIV testing status.

	Tested for HIV				Total		P value
	Yes		No				
	n	%	n	%	n	%	
**Age**							<0.001
15–24	142	14.8	483	41.1	625	29.2	
25–34	255	26.5	229	19.5	484	22.6	
35–44	303	31.5	176	15.0	479	22.4	
45–59	262	27.2	288	24.5	550	25.7	
**Gender**							0.062
Male	433	45.0	577	49.1	1010	47.2	
Female	529	55.0	599	50.9	1128	52.8	
**Education**							<0.001
None and Primary	164	17.2	186	16.0	350	16.6	
Secondary and Vocational	461	48.5	659	56.8	1120	53.1	
University	326	34.3	315	27.2	641	30.4	
**Employment**							<0.001
Unemployed/Housewife/Retired	197	21.2	198	17.0	395	18.9	
Family/ business owner	213	22.9	210	18.1	423	20.2	
Laborer/ Farmer	154	16.5	179	15.4	333	15.9	
Company worker/salaried employee	184	19.8	168	14.5	352	16.8	
Student	45	4.8	295	25.4	340	16.2	
Government employee/Office professional	138	14.8	112	9.6	250	11.9	
**Marital status**							<0.001
Never married	240	25.1	663	56.6	903	42.4	
Married at least once	718	74.9	509	43.4	1227	57.6	
**Residential area**							0.744
Urban	448	46.6	556	47.3	1004	47.0	
Rural	514	53.4	620	52.7	1134	53.0	
**Sexually experienced**							<0.001
No	69	7.2	354	30.1	423	19.8	
Yes	893	92.8	821	69.9	1714	80.2	
**Self-reported history of STIs**							<0.001
No	826	85.9	1096	93.2	1922	89.9	
Yes	136	14.1	80	6.8	216	10.1	
**Personally know someone who has HIV/AIDS**							<0.001
No	772	80.2	1031	87.7	1803	84.3	
Yes	190	19.8	145	12.3	335	15.7	
**HIV risk personalization**							<0.001
Other (unlikely/highly unlikely/not sure)	379	39.4	592	50.3	971	45.4	
Somewhat likely	288	29.9	376	32.0	664	31.1	
Highly likely	295	30.7	208	17.7	503	23.5	
**STI risk personalization**							<0.001
Other (unlikely/highly unlikely/not sure)	399	41.5	605	51.4	1004	47.0	
Somewhat likely	276	28.7	355	30.2	631	29.5	
Highly likely	287	29.8	216	18.4	503	23.5	
**Perceived HIV infection prevalence**							0.013
Other (Decreasing/stable/not sure)	371	38.6	516	43.9	887	41.5	
Increasing	591	61.4	660	56.1	1251	58.5	
**Know where to get HIV test**							<0.001
No	90	9.4	450	38.3	540	25.3	
Yes	872	90.6	726	61.7	1598	74.7	
**Thais are eligible for free and anonymous HIV testing**							0.003
Not correct/ don’t know	480	49.9	662	56.3	1142	53.4	
Correct	482	50.1	514	43.7	996	46.6	
**Received HIV/AIDS related information in the past 12 months**							0.008
No	332	34.5	472	40.1	804	37.6	
Yes	630	65.5	704	59.9	1334	62.4	
**Knowledgeable on HIV transmission, care and treatment**							<0.001
(<9)	367	38.1	673	57.2	1040	48.6	
(≥9)	595	61.9	503	42.5	1098	51.4	

STIs: Sexually Transmitted Infections; AIDS: Acquired Immunodeficiency Syndrome

### Factors associated with HIV testing

Table 2 displays the bivariate and multivariate logistic regression of factors associated with HIV testing. In the adjusted model, having been married (AOR = 2.37; 95% CI = 1.80–3.13), being sexually experienced (AOR = 2.23; 95% CI = 1.59–3.15), history of STIs (AOR = 1.75; 95% CI = 1.25–2.47), knowing someone who has HIV (AOR = 1.38; 95% CI = 1.04–1.84), having personalized HIV risk (Highly likely: AOR = 1.81; 95% CI = 1.02–3.2.1), knowing where to get tested (AOR = 4.57; 95% CI = 3.45–6.05), and being knowledgeable about HIV (AOR = 1.42; 95% CI = 1.14–1.75) were associated with increased odds of being tested for HIV. There was no difference between male and female respondents in terms of HIV testing experience (AOR = 1.19; 95% CI = 0.95–1.49). Respondents who were 45–59 years old were less likely to have been tested for HIV compared to the youngest participants (AOR = 0.68; 95% CI = 0.46–0.99).

**Table 2:** Bivariate and multivariable logistic regression of factors associated with HIV Testing.

	COR	95% CI	P value	AOR	95% CI	p value
**Age**						
15–24	0.32	0.25–0.41	<0.001	1.46	1.00–2.14	<0.049
25–34	1.22	0.95–1.56	0.105	1.91	1.41–2.59	<0.001
35–44	1.89	1.47–2.43	<0.001	1.98	1.49–2.54	<0.001
45–59	1			1		
**Gender**						
Male	1.00			1.00		
Female	1.17	0.92–1.39	0.062	1.19	0.95–1.49	0.127
**Education**						
None and Primary	1			1		
Secondary and Vocational	0.79	0.62–1.01	0.060	1.09	0.81–1.46	0.557
University	1.17	0.90–1.52	0.229	1.34	0.95–1.89	0.086
**Employment**						
Unemployed/Housewife/Retired	1			1		
Family/ business owner	1.01	0.77–1.34		0.91	0.66–1.26	0.602
Laborer/ Farmer	0.86	0.64–1.15	0.891	0.87	0.61–1.23	0.446
Company worker/salaried employee	1.10	0.82–1.46	0.329	1.10	0.77–1.56	0.591
Student	0.15	0.10–0.22	0.513	0.44	0.27–0.72	0.001
Government employee/office professional	1.23	0.90–1.70	<0.001	1.19	0.80–1.76	0.381
**Marital status**						
Never married	1			1		
Married at least once	3.89	3.23–4.69	<0.001	2.37	1.80–3.13	<0.001
**Residential area**						
Urban	1			1		
Rural	1.02	0.86–1.22	0.744	1.10	0.89–1.35	0.347
**Sexually experienced**						
No	1.00			1.00		
Yes	5.58	4.24–7.34	<0.001	2.23	1.59–3.15	<0.001
**Self-reported history of STIs**						
No	1			1		
Yes	2.25	1.68–3.01	<0.001	1.75	1.25–2.47	0.001
**Personally know someone who has HIV/AIDS**						
No	1			1		
Yes	1.75	1.38–2.21	<0.001	1.38	1.04–1.84	0.025
**HIV risk personalization**						
Other (unlikely/highly unlikely/not sure)	1			1		
Somewhat likely	1.19	0.97–1.46	0.080	1.19	0.78–1.80	0.404
Highly likely	2.21	1.77–2.75	<0.001	1.81	1.02–3.21	0.042
**STI risk personalization**						
Other (unlikely/highly unlikely/not sure)	1			1		
Somewhat likely	1.17	0.96–1.44	0.110	0.89	0.59–1.36	0.609
Highly likely	2.01	1.62–2.50	<0.001	0.954	0.54–1.68	0.872
**Perception of HIV prevalence**						
Decreasing/stable/not sure	1			1		
Increasing	1.24	1.04–1.48	0.013	1.03	0.83–1.27	0.779
**Know where to get HIV test**						
No	1			1		
Yes	6.00	4.69–7.68	<0.001	4.57	3.45–6.05	<0.001
**Thais are eligible for free and anonymous HIV testing**						
Not correct/ don’t know	1			1		
Correct	1.29	1.09–1.53	0.003	1.00	0.81–1.23	0.966
**Received HIV/AIDS related information in the past 12 months**						
No	1			1		
Yes	1.27	1.06–1.51	0.008	1.04	0.84–1.29	0.690
**Knowledgeable on HIV transmission, care and treatment**						
(<9)	1			1		
(≥9)	2.16	1.82–2.58	<0.001	1.42	1.14–1.75	0.001

COR: Crude Odds ratio; AOR; Adjusted Odds Ratio; CI: Confidence interval; STIs: Sexually transmitted infections

### Facilitators and reasons for testing for HIV

The most common reasons for HIV testing included routine medical checkup (35.8%) and use of antenatal care (35.3%). The major reason for not being tested included the perception having no (82.8%) or low risk (13.9%). With respect to facilitators of HIV testing, participants named the following reasons as facilitating HIV testing: availability of local testing facilities (32.5%), free testing (31.0%), and anonymous testing (30.6%). (Table 3).

**Table 3:** Facilitators of HIV testing, reasons for testing, and reasons for not testing by gender.

	Men		Women		Total	
	n	%	n	%	n	%
	1010		1128		2138	
**Facilitators of testing**						
Easily accessible, somewhere nearby	314	31.1	381	33.8	695	32.5
Free	319	31.6	344	30.5	663	31.0
Anonymous	328	32.5	326	28.9	654	30.6
Somewhere far away from the neighborhood where no one would know me	25	2.5	50	4.4	75	3.5
Other	24	2.4	27	2.4	51	2.4
**Reasons for testing among the previously tested**	n = 433		n = 529		n = 962	
***Medical checkup or procedure***						
Routine medical checkup	166	38.3	178	33.6	344	35.8
Antenatal care	40	9.2	300	56.7	340	35.3
Blood donation	87	20.1	53	10.0	140	14.6
Surgery or other medical procedures	31	7.2	49	9.3	80	8.3
Pre-marriage/ Family planning	37	8.5	30	5.7	67	7.0
Felt sick	35	8.1	18	3.4	53	5.5
***Personal or relationship reason***						
I was just curious	65	15.0	38	7.2	103	10.7
I engaged in risky behavior	23	5.3	5	0.9	28	2.9
Partner engaged in risky behavior	11	2.5	8	1.5	19	2.0
Partner is infected	0	0.0	0	0.0	0	0.0
***Non-medical reason***						
Application for employment	78	18.0	64	12.1	142	14.8
Insurance application	23	5.3	18	3.4	41	4.3
Military conscription	38		N/A	N/A	38	4.0
Visa application	8	1.8	5	0.9	13	1.4
To be ordained as a monk	7	1.6	N/A	N/A	7	0.7
**Reasons for not testing among the never tested**	n = 577		n = 599		n = 1176	
***Unlikely exposed***						
I have no risk	445	77.1	527	88.3	972	82.8
I have low risk	107	18.5	56	9.4	163	13.9
***Fear of HIV-related stigma***						
Fear of knowing the results	43	7.5	15	2.5	58	4.9
***Access barriers***						
Do not know where to get tested	44	7.6	33	5.5	77	6.6
Cannot afford the test	21	3.6	10	1.7	31	2.6
No time/too busy to go	7	1.2	4	0.7	11	0.9
***Other***						
No use for the results	60	10.4	45	7.5	105	8.9
Afraid of needles	0	0.0	0	0.0	0	0.0

N/A: Not Applicable

### Factors associated with intention to test for HIV

The adjusted model (Table 4) shows a relationship between age and intention to test with younger participants having greater intention to test compared with older participants (AOR = 2.78; 95% CI = 1.94–3.99 for 15–24 years). Sexual experience (AOR = 1.66; 95% CI = 1.25–2.21), history of STIs (AOR = 1.75; 95% CI = 1.26–2.42), knowing someone who has HIV (AOR = 1.38; 95% CI = 1.06–1.81), having personalized STI risk (Highly likely: AOR = 2.34; 95% CI = 1.38–3.94), perception that HIV is increasing in Thailand (AOR: 1.22; 95% CI: 1.01–1.47), and past HIV testing experience (AOR: 1.89; 95% CI = 1.53–2.33) were associated with increased odds of intending to test for HIV.

**Table 4:** Bivariate and multivariable logistic regression of factors associated with intention to test for HIV.

	COR	95% CI	P value	AOR	95% CI	p value
**Age**						
15–24	1.84	1.46–2.33	<0.001	2.78	1.94–3.99	<0.001
25–34	2.02	1.58–2.60	<0.001	1.96	1.46–2.63	<0.001
35–44	2.07	1.61–2.66	<0.001	1.98	1.50–2.61	<0.001
45–59	1			1		
**Gender**						
Male	1			1		
Female	0.82	0.69–0.98	0.029	1.00	0.81–1.23	0.981
**Education**						
None and Primary	1			1		
Secondary and Vocational	1.45	1.13–1.84	0.003	1.13	0.85–1.50	0.337
University	1.32	1.32–1.04	0.039	0.87	0.63–1.20	0.395
**Employment**						
Unemployed/Housewife/Retired	1			1		
Family/ business owner	0.99	0.75–1.31	0.982	1.03	0.76–1.41	0.807
Laborer/ Farmer	1.30	0.97–1.75	0.071	1.26	0.91–1.75	0.157
Company worker/salaried employee	1.53	1.14–2.04	0.004	1.39	0.99–1.93	0.051
Student	1.11	0.83–1.49	0.461	1.03	0.69–1.53	0.861
Governmental/ Professional	1.39	1.01–1.92	0.039	1.60	1.11–2.32	0.012
**Marital status**						
Never married	1			1		
Married at least once	0.95	0.80–1.13	0.632	0.88	0.67–1.14	0.354
**Residential area**						
Urban	1			1		
Rural	0.90	0.76–1.07	0.273	0.91	0.75–1.10	0.347
**Sexually experienced**						
No	1			1		
Yes	1.73	1.39–2.16	<0.001	1.66	1.25–2.21	<0.001
**Self-reported history of STIs**						
No	1			1		
Yes	2.22	1.65–2.98	<0.001	1.75	1.26–2.42	0.001
**Personally know someone who has HIV/AIDS**						
No	1			1		
Yes	1.60	1.26–2.03	<0.001	1.38	1.06–1.81	0.016
**HIV risk personalization**						
Other (unlikely/highly unlikely/not sure)	1			1		
Somewhat likely	1.43	1.17–1.75	<0.001	1.02	0.70–1.48	0.897
Highly likely	2.06	1.66–2.57	<0.001	0.71	0.42–1.21	0.219
**STIs risk personalization**						
Other (unlikely/highly unlikely/not sure)	1			1		
Somewhat likely	1.38	1.13–1.68	0.002	1.19	0.82–1.74	0.341
Highly likely	2.25	1.80–2.80	<0.001	2.34	1.38–3.94	0.001
**Perception of HIV prevalence**						
Decreasing/stable/not sure	1			1		
Increasing	1.34	1.13–1.60	0.001	1.22	1.01–1.47	0.039
**Know where to get HIV test**						
Other*	1			1		
Yes	1.50	1.23–1.83	<0.001	1.11	0.88–1.40	0.371
**Thais are eligible for free and anonymous HIV testing**						
Not correct/ don’t know	1			1		
Correct	1.08	0.91–1.28	0.339	0.98	0.81–1.18	0.840
**Received HIV/AIDS related information in the past 12 months**						
No	1			1		
Yes	1.34	1.13–1.60	<0.001	1.32	1.08–1.61	0.005
**Knowledgeable on HIV transmission, care and treatment**						
(<9)	1			1		
(≥9)	1.21	1.02–1.44	0.023	0.98	0.81–1.19	0.894
**HIV testing experience**						
Never	1			1		
Yes	2.09	1.76–2.49	<0.001	1.89	1.53–2.33	<0.001

COR: Crude Odds ratio; AOR; Adjusted Odds Ratio; CI: Confidence interval; STIs: Sexually transmitted infections

## Discussion

This study examined correlates of HIV testing experience and intention to test using a population-based probability sample in Thailand. We found that less than half of the respondents reported a history of HIV testing and intention to test for HIV in the future.

To date, a few studies have provided data on HIV testing rates among the Thai general population. In the 2006 National Sexual Behavior Survey, 48% of the 6048 participants reported having been tested for HIV [5]. According to the 2009 Fertility Survey, among 37,511 Thai women aged 15–59 years, 15% reported that either they or their spouse had received premarital family planning counseling, and within that group 20.9% had a premarital HIV test as part of the counseling [20]. More recent population data on the prevalence of HIV testing among Thai general population does not exist, making it hard to assess testing trends over time, as well as the relevance of the correlates of HIV testing documented in this study with regards to the current context of Thailand. Recent data on the UNAIDS 90-90-90 targets indicate that for Thailand, 94% of people living with HIV know their HIV status [16]. While this is an indication of improved HIV testing coverage in Thailand, it is unclear to what extent this metric translates knowledge of HIV status in the general population given that no recent HIV testing data exists for the general population. Thailand has successfully piloted a number of strategies to increase access to HIV testing particularly for key populations, including MSM, female sex workers (FSW), and PWID. These strategies include provision of free HIV testing and same-day results, the use of lay providers to deliver HIV testing services, implementation of community-based HIV testing and counseling to expand outreach [30, 31], as well as passage of recent regulation by Thailand’s Food and Drug Administration authorizing pharmacies to sell self-testing kits [32]. According to recent data from Thailand, HIV testing coverage, defined as receipt of a test in the last 12 months, was only 29% among MSM, 58% among FSW, and 61% among PWID in 2018. Compared to data from 2008–2009, HIV testing coverage among MSM, FSW, and PWID was respectively 21.3%, 35.2%, and 59.7% [30]. It is difficult to predict to what extent HIV testing prevalence has evolved in the Thai general population since 2012, making it crucial to conduct research to capture the current situation of HIV testing and related factors in the general population. Our study has the merit of serving as baseline data for future research in this area.

We found that sexual experience and history of STIs were associated with HIV testing experience and intention to accept HIV testing. This could reflect the increased demand for sexual and reproductive health services (SRH) upon initiating sexual activity (for the association with sexual experience) and positive attitudes toward HIV testing (for the association with intention to test). It is possible that these associations are mediated by perceptions of HIV or STIs risk. Previous research has linked high perception of HIV risk to HIV testing experience and intention to uptake HIV testing [19, 23, 33]. In our study, however, variables that captured diverse dimensions of risk perception were independently associated with either past HIV testing experience or intention to test, or both. For example, individuals who perceived themselves to be at high risk for HIV and those who personally knew someone infected with HIV were more likely to report having been tested for HIV. Similarly, perceiving high self-risk for STIs, an increase in HIV prevalence in Thailand, and personally knowing someone infected with HIV made participants more likely to have intention to test for HIV. Furthermore, the perception that one had “no risk” or “low risk” for HIV was cited as one of the main reasons for not testing in our study, suggesting that HIV risk personalization could be a determining factor for HIV testing. Interventions that emphasized risk personalization significantly improved knowledge, attitude, and behavior related to HIV and STIs among high school students in Japan [34].

There were also discrepancies between factors associated with past HIV testing experience and intention to test for HIV. For example, knowledge of where to get tested for HIV was significantly associated with having been tested for HIV. However, this factor had no association with intention test. This implies that people became aware of HIV testing locations as a result of their previous HIV testing experience rather than the other way round. Similarly, having a history of marriage was associated with past HIV testing experience, but not with intention to test. The relationship between marriage and HIV testing could be affected by antenatal care. In Thailand, antenatal care includes free HIV testing for women and their partners [35]. Most of the women in our study reported having been tested as part of antenatal care. It is important to note that HIV screening through antenatal care leaves out a large proportion of sexually active young girls who are not pregnant. With sexual debut getting earlier, [3], and consequently, the widening interval between sexual initiation and the use of antenatal care services (generally after marriage in the Thai context), it is crucial to develop innovative strategies to encourage HIV testing among young women outside the context of antenatal care. Encouraging the husbands or partners of pregnant women to get tested for HIV, individually or as a couple, is another strategy that could help increase testing rates in the young male population.

Consistent with previous research [36, 37], there was an association between past HIV testing experience and intention to test, indicating that efforts to get people tested for the first time would be a significant investment HIV testing attitudes and behavior in the future. It is worth noting that there was no association between awareness of free anonymous testing for Thai nationals with HIV testing experience or intention to test; only less than half of our respondents were aware of their eligibility to receive free and anonymous HIV testing. In contrast, a substantial proportion of respondents reported that free (31.0%) and anonymous (30.6%) HIV testing would make it easier for them to go for an HIV test. This reveals a gap in information regarding HIV testing among the general Thai population and that an appropriately designed HIV/AIDS campaign to disseminate accurate information regarding HIV testing might likely increase the number of people testing for HIV. In support of this, respondents who received HIV/AIDS education in the last year were more likely to report intention to test for HIV.

We found that age was associated with HIV testing experience; particularly those between 25–44 years were more likely to test for HIV than 44–59 year olds. Age was also associated with intention to test for HIV, with younger age groups being more likely to consider a future HIV testing opportunity. Participants who were 15–24 years old were most likely to have intention to test for HIV in our study. At the global level, this age group (15–24 years) accounts for 35% of global new HIV infection occurring annually [38]. There is a window of opportunity for tailored interventions to improve the HIV testing rate among the younger Thai population through strategies such as provider-initiated HIV testing and counseling and HIV self-testing. The Thai Food and Drug Administration approved over-the-counter sales of HIV self-testing kits earlier this year. A systematic review found that HIV self-testing increased uptake and frequency of testing particularly among those who are at risk [39]. For such interventions to be effective, structural barriers to accessing HIV testing services for adolescents as well as limited HIV/AIDS related information would need to be addressed. In 2012, one such structural barrier was removed when Thailand did away with parental consent for those under 18 who wanted to get tested for HIV. This has likely improved the landscape of HIV testing in Thailand [14].

This study is in not without limitations. This is a cross-sectional study, hence, we cannot draw causal inferences from the documented associations. There is also the possibility of recall bias and the sensitive nature of the study may have increased socially desirable answers. Efforts were made to minimize these biases through the use a self-administered questionnaire through an internet-enabled tablet. In addition, the results are based on data collected in 2012, which might not reflect current trends in HIV testing in Nonthaburi province. Considering that access to HIV testing and level of HIV knowledge in the population may have improved since 2012 as a result of HIV programs, it is unclear which correlates of HIV testing and intention to test are relevant in the current context of Thailand. However, given the dearth of population-based data available, our findings could serve as a benchmark for future population-based research on HIV testing in Thailand. This study has important strengths that are worth mentioning. This study was designed to maximize methodological validity. Sampling was by means of multistage probability sampling at a provincial scale with extensive mapping and efforts were made to visit sampled households multiple times if participants were not at home. These efforts yielded a high overall response rate of 85.5% [3].

## Conclusions

We found that a low proportion of the respondents reported a history of HIV testing and intention to test for HIV. There is urgent need to develop strategies that reach out to the general population. Interventions will need to integrate risk personalization approaches with diffusion of accurate and culturally-adapted information related to HIV infection and HIV testing in Thailand.

## Supporting information

S1 Table Knowledge on HIV transmission, care and treatment. (DOC)

S2 Table Sociodemographic and behavioral characteristics of the participants by intention to test for HIV. (DOC)

S1 Dataset (SAV)
