# Supplementary material for: Factors associated with HIV testing and intention to test for HIV among the general population of Nonthaburi Province, Thailand
Source: PLoS One. 2020 Aug 14;15(8):e0237393. doi: 10.1371/journal.pone.0237393 (PMC7428091; doi:10.1371/journal.pone.0237393)
Supplement: S1 Table — (DOC) [file pone.0237393.s001.doc]

**S1 Table.** Knowledge on HIV transmission, care and treatment

|  | True | False | Don’t know |
| --- | --- | --- | --- |
| HIV can be transmitted by mosquito bites |  |  |  |
| HIV can be transmitted through a mouth to mouth kiss |  |  |  |
| If a mother is infected with HIV there is a chance of transmission to the infant |  |  |  |
| HIV can be transmitted through sharing a needle or syringe with a person infected with HIV |  |  |  |
| It is not safe to share meals with a person infected with HIV |  |  |  |
| Even unprotected, there is zero chance for HIV transmission during oral sex |  |  |  |
| HIV transmission can be prevented by using condom correctly every time during sexual intercourse |  |  |  |
| Being faithful to only one uninfected partner who does not have other partner is a way to reduce HIV infection |  |  |  |
| It is possible that a healthy looking person has HIV |  |  |  |
| There is a vaccine to prevent HIV |  |  |  |
| HIV/AIDS can now be completely cured with medicine |  |  |  |
| HIV infection can be detected by a test within a few days after infection |  |  |  |
