# Supplementary material for: Factors associated with HIV testing and intention to test for HIV among the general population of Nonthaburi Province, Thailand
Source: PLoS One. 2020 Aug 14;15(8):e0237393. doi: 10.1371/journal.pone.0237393 (PMC7428091; doi:10.1371/journal.pone.0237393)
Supplement: S2 Table — (DOC) [file pone.0237393.s002.doc]

**S2 Table. Sociodemographic and behavioral characteristics of the participants by intention to test for HIV**

|  | **Intention to test for HIV** | | | |  |  |  |
| --- | --- | --- | --- | --- | --- | --- | --- |
|  | Yes | | Else | | Total | | P value |
|  | n | % | n | % | n | % |  |
| **Age** |  |  |  |  |  |  | <0.001 |
| 15-24 | 318 | 30.8 | 306 | 27.7 | 624 | 29.2 |  |
| 25-34 | 258 | 25.0 | 226 | 20.5 | 484 | 22.6 |  |
| 35-44 | 258 | 25.0 | 221 | 20.0 | 479 | 22.4 |  |
| 45-59 | 198 | 19.2 | 352 | 31.9 | 550 | 25.7 |  |
| **Gender** |  |  |  |  |  |  | 0.029 |
| Male | 513 | 49.7 | 497 | 45.0 | 1010 | 47.3 |  |
| Female | 519 | 50.3 | 608 | 55.0 | 1127 | 52.7 |  |
| **Education** |  |  |  |  |  |  | 0.011 |
| No and Primary | 145 | 14.2 | 205 | 18.8 | 350 | 16.6 |  |
| Secondary  and Vocational | 567 | 55.5 | 553 | 50.8 | 1120 | 53.1 |  |
| University | 309 | 30.3 | 331 | 30.4 | 640 | 30.3 |  |
| **Employment** |  |  |  |  |  |  | 0.012 |
| Unemployed/Housewife/Retired | 174 | 17.2 | 221 | 20.4 | 395 | 18.9 |  |
| Family/ business owner | 186 | 18.4 | 237 | 21.9 | 423 | 20.2 |  |
| Labor/ Farmer | 169 | 16.7 | 164 | 15.2 | 333 | 15.9 |  |
| Company worker/salary man | 192 | 19.0 | 159 | 14.7 | 351 | 16.8 |  |
| Students | 181 | 16.7 | 159 | 15.7 | 340 | 16.3 |  |
| Governmental/ Professional | 131 | 13.0 | 119 | 11.0 | 250 | 12.0 |  |
| **Marital status** |  |  |  |  |  |  | 0.632 |
| Never married | 441 | 42.9 | 461 | 41.9 | 902 | 42.4 |  |
| Ever married | 587 | 57.1 | 640 | 58.1 | 1227 | 57.6 |  |
| **Residential area** |  |  |  |  |  |  | 0.273 |
| Urban | 497 | 48.2 | 506 | 45.8 | 1003 | 46.9 |  |
| Rural | 535 | 51.8 | 599 | 54.2 | 1134 | 53.1 |  |
| **Ever had sex** |  |  |  |  |  |  | <0.001 |
| No | 158 | 15.3 | 264 | 23.9 | 422 | 19.8 |  |
| Yes | 874 | 84.7 | 840 | 76.1 | 1714 | 80.2 |  |
| **Self-reported history of STIs** |  |  |  |  |  |  | <0.001 |
| Never | 890 | 86.2 | 1031 | 93.3 | 1921 | 89.9 |  |
| Ever | 142 | 13.8 | 74 | 6.7 | 216 | 10.1 |  |
| **Personally know someone who has HIV/AIDS** |  |  |  |  |  |  | <0.001 |
| Else | 220 | 21.3 | 320 | 29.0 | 540 | 25.3 |  |
| Yes | 812 | 78.7 | 785 | 71.0 | 1597 | 74.7 |  |
| **HIV risk personalization** |  |  |  |  |  |  | <0.001 |
| Else | 401 | 38.9 | 570 | 51.6 | 971 | 45.4 |  |
| Somewhat likely | 333 | 32.3 | 330 | 29.9 | 663 | 31.0 |  |
| Highly likely | 298 | 28.9 | 205 | 18.6 | 503 | 23.5 |  |
| **STIs risk personalization** |  |  |  |  |  |  | <0.001 |
| Else | 414 | 40.1 | 590 | 53.4 | 1004 | 47.0 |  |
| Somewhat likely | 310 | 30.0 | 320 | 29.0 | 630 | 29.5 |  |
| Highly likely | 308 | 29.8 | 195 | 17.6 | 503 | 23.5 |  |
| **HIV infections increasing in Thailand** |  |  |  |  |  |  | 0.001 |
| Else | 390 | 37.8 | 497 | 45.0 | 887 | 41.5 |  |
| Increasing | 642 | 62.2 | 608 | 55.0 | 1250 | 58.5 |  |
| **Know where to get HIV test** |  |  |  |  |  |  | <0.001 |
| Other | 220 | 21.3 | 320 | 29.0 | 540 | 25.3 |  |
| Yes | 812 | 78.7 | 785 | 71.0 | 1597 | 74.7 |  |
| **knowledge of Thai eligibility for free and anonymous HIV testing** |  |  |  |  |  |  | 0.339 |
| Else | 540 | 52.3 | 601 | 54.4 | 1141 | 53.4 |  |
| Correct | 492 | 47.7 | 504 | 45.6 | 996 | 46.6 |  |
| **Had received HIV/AIDS related information in the past 12 months** |  |  |  |  |  |  | 0.001 |
| No | 351 | 34.0 | 453 | 41.0 | 804 | 37.6 |  |
| Yes | 681 | 66.0 | 652 | 59.0 | 1333 | 62.4 |  |
| **Knowledge on HIV transmission, care and treatment** |  |  |  |  |  |  | 0.023 |
| (<9) | 476 | 46.1 | 564 | 51.0 | 1040 | 48.7 |  |
| (≥9) | 556 | 53.9 | 541 | 49.0 | 1097 | 51.3 |  |
| **HIV testing experience** |  |  |  |  |  |  | <0.001 |
| Never | 471 | 45.6 | 705 | 63.8 | 1176 | 55.0 |  |
| Ever | 561 | 54.4 | 400 | 36.2 | 961 | 45.0 |  |

STIs: Sexually transmitted infections
